# Influence of the catchment area use on the water quality in the Utrata River

**DOI:** 10.1007/s10661-022-09821-z

**Published:** 2022-02-10

**Authors:** Katarzyna Dębska, Beata Rutkowska, Wiesław Szulc

**Affiliations:** Institute of Agriculture, Independent Department of Agriculture Chemistry, Warsaw, University of Life Sciences, Nowoursynowska 166, 02-787 Warsaw, Poland

**Keywords:** Total phosphorus, Nitrate nitrogen, Ammonium nitrogen, River pollution, Utrata River, Biogenic substances

## Abstract

The present paper discusses the impact of land use and seasons on the concentration of nutrients in the waters of the Utrata River (Pruszków Poviat, Mazowieckie Voivodeship) from April 2018 to March 2019. The pollution of rivers by nutrients is a major problem for society. Surface water is a source of drinking water, water used for industrial and agricultural purposes. With the increasing pollution of rivers, the purification process for these purposes becomes more expensive and more challenging. To assist in carrying out activities aimed at reducing the inflow of biogenic substances into large river systems and then down to the Baltic Sea, we analyzed the spatial and temporal dynamics of loads from the entire Utrata River catchment area. We divided the entire catchment area into three impact zones: grasslands and wastelands, urbanized areas, and agricultural land and examined changes in nutrient concentrations (total phosphorus, nitrate nitrogen, ammonium nitrogen) in each of the zones. The results were statistically processed using the 1-factor ANOVA method with the *p*-value of significance below 0.05. Research indicates an increase in the concentration of total phosphorus and nitrogen forms down the course of the river in urban and agricultural areas with persistently low concentrations of these biogenic substances in grasslands.

## Introduction

**Fig. 1:**
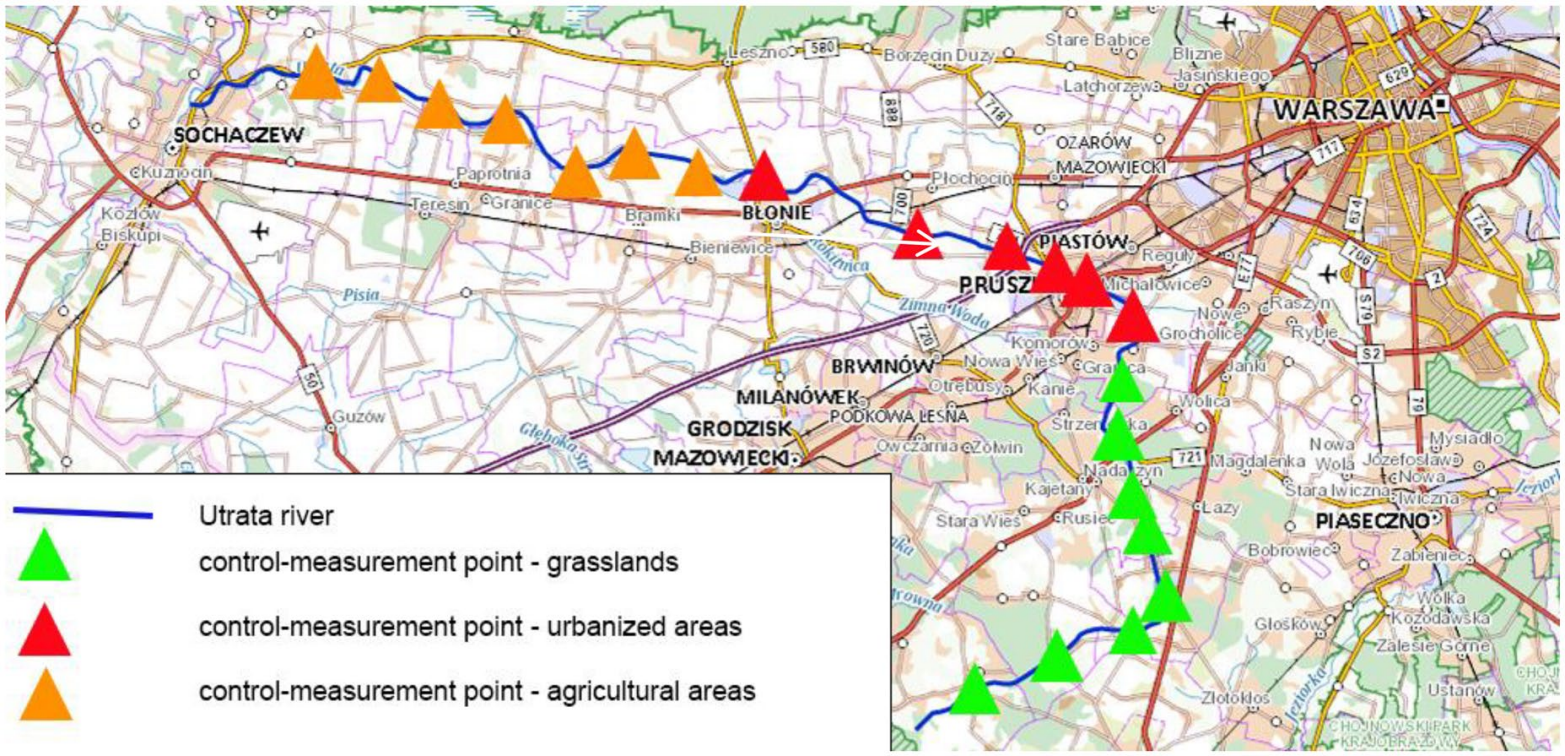
Study area: location control-measurement point in the Utrata River (https://mapy.geoportal.gov.pl/imap/Imgp_2.html?gpmap=gp0; access 11 Feb 2021)

**Fig. 2:**
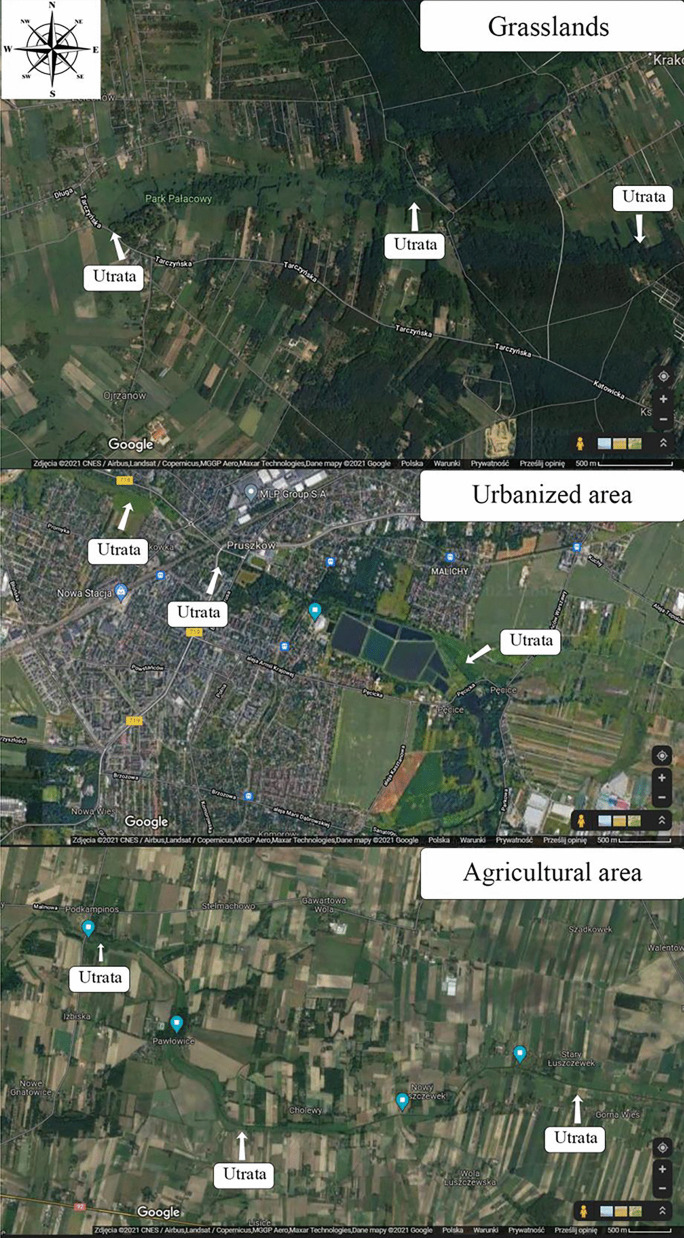
Study area: land use in the Utrata River catchment area (https://www.google.pl/maps/@52.0917827,20.757677,10z/data=!5m1!1e4; access 11 Mar 2021)

The quality of the water flowing out of the catchment area of a river depends primarily on the form of land development (Fig. 1). Other factors on which water quality depends are climate, weather conditions, the presence of buffer zones, and size and location of the catchment area (Lúcia et al., 2020). The in-depth knowledge of the sources of pollution and the dynamics of its travel in the riverbed are crucial for the preservation and improvement of river water quality. The analysis of water from the entire course of the river, taking into account, for example, the uses of the catchment area (agricultural use areas, urbanized areas, and grasslands) gives us such a possibility (Fig. 2) (Schmalz & Kruse, 2019). Biogenic pollutants in Polish rivers flow into the Baltic Sea, causing its eutrophication. Polluted rivers are responsible for 100% of the phosphorus present in the Baltic Sea and some 20–30% of its nitrogen (Bartnicki, 2019).

**Fig. 3:**
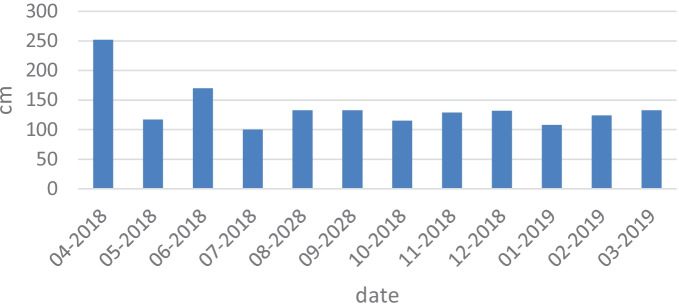
Water level in the Utrata River on the measurement days ( source: https://dane.imgw.pl/data/dane_pomiarowo_obserwacyjne/)

Research conducted by Paule-Mercado et al. (2017), Cavalcante et al. (2017), and Rodrigues et al. (2018) demonstrates that the link between the quality of water in rivers and the type of catchment used (urbanized and agricultural land) varies, depending on the terrain, region, weather, and climatic conditions. Therefore, it proves necessary to conduct comprehensive research in various regions in order to fully understand this relationship (Baker, 2006). What was noticed in recent years is the changing dynamics of pollution in surface waters, resulting from global warming, which results in more frequent droughts and floods that lead to changes in the condition of river waters and changes in pollutant concentrations (Fig. 3) (Dzhamalov et al., 2019).

The waters in agricultural areas contain high concentrations of biogenic substances such as total phosphorus, nitrate nitrogen, and ammonium nitrogen, the sources of which are organic and mineral fertilizers (Fierro et al., 2017). High local concentrations of phosphorus may also be the result of municipal pollution (Shen et al., 2020).

An increase in nitrate nitrogen concentration may also be triggered by agriculture, especially in the lowlands, in the lower parts of the river, where organic matter is nitrified and fertilizer components are leached (Mingming et al., 2019).

Ammonium nitrogen, next to nitrate nitrogen, is another indicator of surface water pollution. The concentration of ammonium nitrogen depends on the season and climatic and atmospheric conditions and usually increases in the lower parts of the river. The main sources of ammonium nitrogen in rivers are point sources, e.g., sewage treatment plants and surface sources — surface runoffs from farmlands. However, the sources of pollution and the factors affecting its concentration in rivers may be unique to each case study (Armstrong et al., 2012). In recent years, decreasing concentrations of ammonium nitrogen in river waters have been observed, which is related to the improvement of wastewater management (Wang et al., 2016). Studies demonstrate that long-term elevated concentrations of ammonium nitrogen in rivers negatively impact microbiological life, also resulting in the possible loss of benthic diversity of invertebrates (Shi et al., 2019; Hampel et al., 2017).

The impact of climate change, summer droughts, and floods has, to the present date, been evaded in research; however, some studies demonstrated that droughts have a significant effect on temperature and dissolved oxygen content but do not affect the concentration of biogenic substances (Zwolsman & van Bokhoven, 2007).

The objective of our research effort is to analyze the dynamics of pollution and identify the sources of pollution within the Utrata River catchment area.

## Methods

The Utrata River is 78 km long and its catchment area amounts to approximately 702 km^2^. It flows through the Mazowieckie Voivodeship and has its source in Ojrzanów and its outlet in Sochaczew (where it becomes a tributary of the Bzura River). The exposure of the Utrata River to pollution comes from the following sources: fish ponds, sewage treatment plants, heat and power plants, landfills, and progressive urbanization (Wojtkowska, 2006). Agriculture forms a very significant source of pollution entering the river. A considerable proportion of the catchment area is covered by black earth (fertile soils), which makes agriculture in this area highly developed and specialized (Rydałowski et al., 2008).

**Fig. 4:**
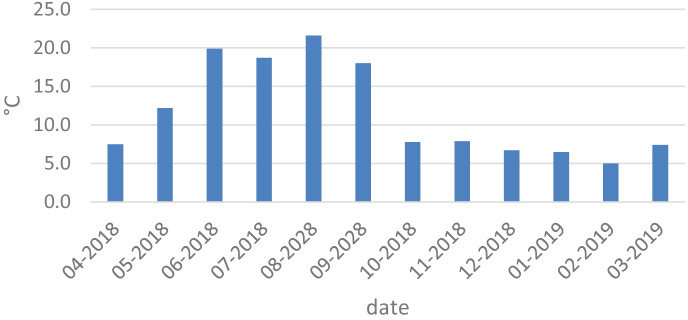
Air temperature detected on the measurement days (https://dane.imgw.pl/data/dane_pomiarowo_obserwacyjne/)

**Fig. 5:**
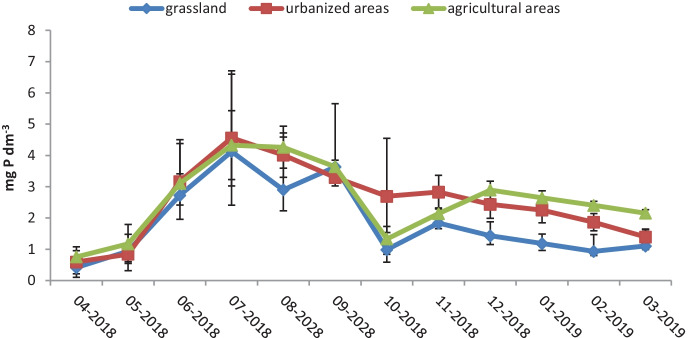
Concentration of total phosphorus in the Utrata River during the months of April 2018–March 2019

**Fig. 6:**
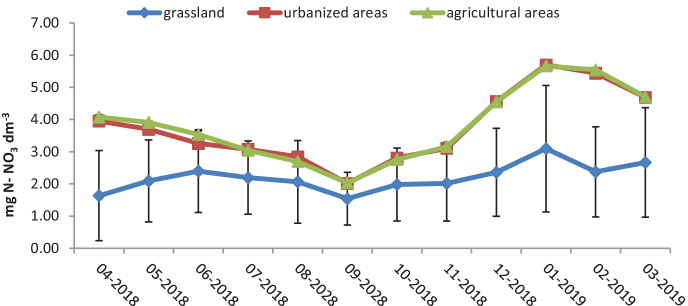
Concentration of nitrate nitrogen in the Utrata River in the months of April 2018–March 2019

Water samples for analyses were collected monthly from April 2018 to March 2019 at 21 sampling locations located along the entire river course (Figs. 4, 5, and 6). The study of the river was scheduled for 1 year in order to observe the influence of the season on changes in concentrations of the nutrients. The sampling points were located in areas of different land use. The Utrata River was divided into 3 areas: grasslands from the river source to the 26th km, urbanized areas from the 26th to the 49th km, and agricultural areas from the 49th km to the river outlet. The river measurements were created basing on field observations and using the QGIS software. Samples 1–8 came from grasslands, 9–14 from urbanized areas, and 15–21 from agricultural areas. Two hundred fifty-two samples were collected.

We tested the concentrations of total phosphorus with the use of the spectrophotometric method with ammonium molybdate (according to PN-EN ISO 6878: 200) and ammonium and nitrate nitrogen with the use of flow colorimetry (according to PN-EN ISO 11732: 2007)

The results were processed using the 1-factor ANOVA method with the *p*-value of significance below 0.05 (Table 1).

**Table 1:** Permissible values in the first and second water quality classes of total phosphorus and ammonium and nitrate nitrogen in Utrata River according to the Polish Regulation of the Minister of Maritime Economy and Inland Navigation

**Pollutants**	**Class**	**Values**
N-NO_3_ (mg/dm^3^)	I water quality class	1.1
	II water quality class	2.0
N-NH_4_ (mg/dm^3^)	I water quality class	0.25
	II water quality class	0.738
Total P (mg/dm^3^)	I water quality class	0.17
	II water quality class	0.33

The measurement results were compared with the Polish Regulation of the Minister of Maritime Economy and Inland Navigation on 11 October (2019) on the classification of ecological condition, ecological potential, chemical condition, and the method of classification of the state of surface water bodies, as well as environmental quality standards for priority substances.

## Results

The average concentration of total phosphorus in the waters of the Utrata River amounted to 2.15 mg P/dm^3^, significantly exceeding the permissible values for class I and II water quality (according to the Polish Regulation of the Minister of Maritime Economy and Inland Navigation), which are 0.17 and 0.33 mg P/dm^3^, respectively. The lowest average concentration of total phosphorus in waters of the river was measured in spring on grasslands (sampling locations 1 to 8), and it amounted to an average of 0.78 mg P/dm^3^, while the highest concentration of total phosphorus was observed in summer in agricultural and urbanized areas (with 3.9 and 3.91 mg P/dm^3^).

The difference in average values of total phosphorus concentration in urbanized areas as compared to grasslands was 0.64 mg P/dm^3^, while the average difference in concentration in agricultural areas compared to urbanized areas amounted to just 0.07 mg P/dm^3^.

The concentration of phosphorus gradually increased from the first measurement month (April) to July, when it reached the highest values, regardless of the type of land use. In the summer period (June–September), the phosphorus concentrations remained at a very high level. In October, there was a sharp decrease in phosphorus concentration in grasslands and agricultural areas, while at the same time high values were still recorded in urbanized areas. Regardless of the land use, in the winter months (December–February), the concentrations of phosphorus dropped.

The average concentration of nitrate nitrogen was 3.06 mg N-NO_3_/dm^3^, and it fell outside the range of class II water quality (according to the Polish Regulation of the Minister of Maritime Economy and Inland Navigation). Only in grasslands was the average concentration of nitrate nitrogen (2.2 mg N-NO_3_/dm^3^) within the range for the class II water quality. The average concentration of nitrate nitrogen in urbanized areas was 3.76 mg N-NO_3_/dm^3^, while in agricultural areas, it amounted to 3.81 mg N-NO_3_/dm^3^; both of these values significantly exceed the permissible values for class I and II water quality, which are 1.1 and 2.0 mg N-NO_3_/dm^3^, respectively (Table 2).

**Table 2:** Statistical analysis (one-way ANOVA)

***Y***	***X***	***n***	***p*-value**
N-NO_3_ (mg/dm^3^)	Land use	252	2.1E−25
	Air temperature	252	1.87E−16
	Season of the year	252	9.5E−17
	Water level	252	4.41E−11
N-NH_4_ (mg/dm^3^)	Land use	252	6.00E−37
	Season of the year	252	1.9E−08
	Air temperature	252	2.51E−08
	Water level	252	3.30E−05
Total P (mg/dm^3^)	Land use	252	1.5E−07
	Season of the year	252	1.1E−23
	Air temperature	252	9.93E−23
	Water level	252	1.60E−18

The lowest average concentration of nitrate nitrogen was recorded in autumn in grasslands and was 1.85 mg N-NO_3_/dm^3^, while the highest average concentration was observed in winter in agricultural and urbanized areas (5.23 and 5.25 mg N-NO_3_/dm^3^, respectively).

The average excess in nitrate nitrogen concentrations in urbanized areas as compared to grasslands was 1.56 mg N-NO_3_/dm^3^, while its average excess in concentration in agricultural areas compared to urbanized areas amounted to 0.04 mg N-NO_3_/dm^3^.

The average concentration of ammonium nitrogen in the Utrata River was 0.81 mg N-NH_4_/dm^3^, and it exceeded the permissible threshold values for water quality classes I and II (according to the Polish Regulation of the Minister of Maritime Economy and Inland Navigation). The average concentration of ammonium nitrogen was the lowest in grasslands, amounting to 0.51 mg N-NH_4_/dm^3^ (the lowest observed averaged concentration was 0.27 mg N-NH_4_/dm^3^ and it was within the limits of the class II water quality).

The average concentration of ammonium nitrogen in urbanized and agricultural areas did not differ significantly and amounted to 1.07 mg N-NH_4_/dm^3^ for the urbanized areas and 1.08 mg N-NH_4_/dm^3^ for the agricultural ones. The highest average concentration of this form was recorded in summer in agricultural areas, where it amounted to 1.4 mg N-NH_4_/dm^3^, significantly exceeding the permissible value for class II water quality (0.4 mg N-NH_4_/dm^3^).

The average excess in ammonium nitrogen concentrations in urbanized areas as compared to grasslands was 0.56 mg N-NH_4_/dm^3^, while its average excess in concentration in agricultural areas compared to urbanized areas amounted to 0.01 mg N-NH_4_/dm^3^.

The concentration of ammonium nitrogen in grasslands was lower than that in urbanized and agricultural areas (with the average value in green areas of 0.51 mg N-NH_4_/dm^3^ and averages in urbanized and agricultural areas of 1.07 and 1.08 mg N-NH_4_/dm^3^, respectively); only for September did we record an abrupt shift in the concentration of ammonium nitrogen in green areas.

Statistical analysis proved a significant impact on the season and the way land was used on all analyzed indicators, but it failed to prove the influence of the water level or weather conditions.

## Discussion

The cause behind increased phosphorus concentrations in rivers in urbanized areas can be runoffs from sewage treatment plants and storm drain systems, and it has been proven in many studies (Hu et al., 2019; Charlton et al., 2017). In October, we can observe higher concentrations of phosphorus in urbanized areas, as compared to agricultural and grassland areas, which may be related to the inflow to the Utrata from the highly polluted Raszynka River. Raszynka could deliver a significant load of phosphorus, which becomes diluted further downriver. The significant effect of the sewage treatment plants on the phosphorus concentrations in rivers was also observed by Yuan et al. (2019).

The increased inflow of total phosphorus in agricultural areas in summer is associated with rainfall washing away phosphorus fertilizers from the soil (Ryberg, 2017; Boardman et al., 2019), which, as the terrain drops, then gets into river valleys (Wu & Lu, 2019).

The concentration of nitrate ions in the waters of the Utrata River increased in the urbanized area, and this shift in concentration may be related to the discharge of water from the sewage treatment plants. On the other hand, the increase in the concentration of nitrate ions in agricultural areas may be related to leaching nitrogen fertilizers from the soil (Torma et al., 2019). The presence of nitrate ions in grasslands may indicate leaky septic tanks located in the catchment area (Górski et al., 2019). The highest concentrations of nitrate ions were recorded in winter, and Dolgov and Koronkevich (2019) also report that the greatest leaching of nitrate ions from agricultural areas occurs between December and February.

The high concentration of ammonium ions in the water of the Utrata River may indicate its severe pollution with commercial and industrial sewage (Agca & Doğan, 2019). A large change in the concentration of ammonium ions in September in grasslands may be associated with a one-time pollution from a single source, e.g., discharge of sewage straight into the river, and these pollutants were diluted further downstream.

A large change in the concentration of biogenic substances is related to the presence of a sewage treatment plant, the discharge of which ends up in the waters of the Utrata River (sampling locations 5 and 6). Even properly operating sewage treatment plants fail to remove 100% of the biogenic pollutants (Bawiec, 2018).

Wastewater treatment plants located in the vicinity of the Utrata River significantly affect its flow and chemical condition. Research proves that for over 50 years, the growing urbanization of areas (and the resulting fragmentation of the area), human activity, and climate change have significantly influenced the chemical condition of the water in the upper and middle courses of the Utrata River (Somorowska & Łaszewski, 2019).

A large inflow of pollutants may also be attributed to the unused fish ponds upstream of Walendow, which is probably caused by very substantial contamination of the ponds with biogenic substances that get into the river from the bottom sediments. On the other hand, the increase in the concentration of pollutants in location no. 9 could have occurred as a result of the presence of fish ponds in Pęcice and the influx of pollutants from the Raszynka River to the researched Utrata River. Kieu et al. (2020) also observed a high content of phosphorus and nitrogen forms in fish ponds.

Raszynka is a river with low water quality, and research carried out by Burzyńska published in (2019) demonstrated high concentrations of nitrogen and phosphorus in its waters. The waters of the Raszynka River were the most polluted in its lower course at the mouth, which may explain the increase in nutrient concentration in Utrata (sampling location 9). A 2015 research also confirmed high levels of pollution in the Raszynka River (Burzyńska, 2016).

The high concentration of nitrate ions, ammonium ions, and total phosphorus in combination with the high temperature of that river (Łaszewski, 2016) and the progressive urbanization may have led to the eutrophication process, which can be observed more and more often in river waters (Hutchins & Hitt, 2019).

**Fig. 7:**
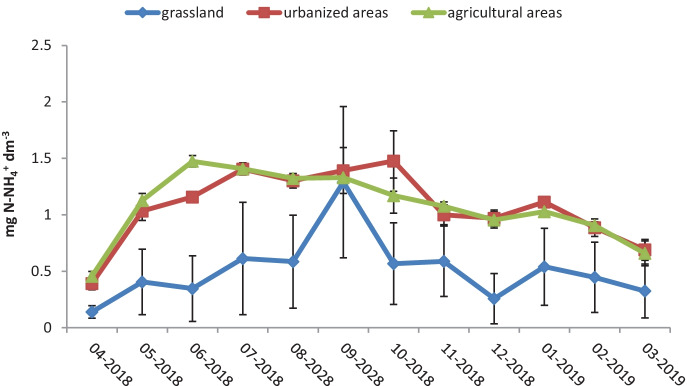
Concentration of ammonium nitrogen in the Utrata River in the months of April 2018–March 2019

The analysis carried out on the river measurement results failed to prove a statistically significant influence of water levels or air temperature on the concentration of biogenic substances in Utrata (Fig. 7). The negative effect of high air temperature on the overall water quality of rivers was proven by Ozaki et al. (2003), but the effect is limited to changes in concentrations of dissolved oxygen, COD, and water temperature. No data would seem to indicate an effect of air temperature on the concentration of biogenic substances in rivers.

## Conclusion

The waters of the Utrata River were of poor quality owing to the high concentration of ammonium ions, nitrate ions, and total phosphorus. The most polluted part of the river was its lower course, which was dominated by agricultural land development; the least polluted were the waters from the upper part of the river, where wastelands, forests, and low levels of urbanization prevailed.

The Utrata River is polluted with nitrate and ammonium nitrogen in urbanized and agricultural areas.

We recorded a strong correlation between the concentration of the tested biogenic substances and the season of the year, and at the same time, no influence of air temperature and water level on the pollution.
